# Knitting the Threads of Silk through Time: Behçet's Disease—Past, Present, and Future

**DOI:** 10.1155/2017/2160610

**Published:** 2017-09-10

**Authors:** Fahd Adeeb, Austin G. Stack, Alexander D. Fraser

**Affiliations:** ^1^Department of Rheumatology, University Hospital Limerick, Limerick, Ireland; ^2^Graduate Entry Medical School, University of Limerick, Limerick, Ireland; ^3^Health Research Institute, University of Limerick, Limerick, Ireland; ^4^Department of Medicine, Universiti Teknologi MARA, Sungai Buloh, Selangor, Malaysia; ^5^Department of Nephrology, University Hospital Limerick, Limerick, Ireland

## Abstract

Behçet's disease (BD) is a chronic relapsing vasculitis that affects vessels of all types and sizes with a broad spectrum of phenotypic heterogeneity and complex immunopathogenesis. Efforts by the scientific community to resolve the unmet needs of BD and gaps in our knowledge have been hampered by considerable challenges that primarily relate to the rare nature of the disease in many parts of the world and its heterogeneity. Controversies remain in many aspects of the disease including the diagnostic criteria, immunopathogenesis and biomarker discovery, geographical variation, and therapeutic considerations. In this review, we highlight recent advances in our scientific understanding of BD, shed new insights into diagnostic and treatment strategies, and discuss residual gaps in our knowledge that will serve as the basis for current and future research.

## 1. Introduction

Behçet's disease (BD), also known as the* Silk Road disease* [1], was first described in the Cyclades island of Kos by Hippocrates in his writings* Third Book of Endemic Diseases* [2, 3] but remained in relative obscurity for more than two millennia until 1937 when a Turkish dermatologist in Istanbul, Hulusi Behçet, described a trisymptom complex of recurrent aphthous stomatitis, genital ulcers, and iritis in three native Eastern Mediterranean patients and posited a new clinical syndrome [4].

Previously described among populations extending around the ancient Silk Road, a trading route that stretched from the Iberian Peninsula on the southwestern tip of Europe, across Iran, Iraq, and Syria of the Middle East to the Far East, BD has a global distribution and can be seen throughout the world including countries further north and south of the equator (≥60°). The principle morbidity of BD relates to its vascular, ophthalmic, and neurological complications, and if left untreated, it may lead to blindness and death.

In this review article, we explore recent studies that have substantially contributed to our understanding of BD in recent years and have provided new insights into the epidemiology, etiology, immunopathogenesis, clinical manifestations, classification criteria, and management strategies of BD as well as identifying areas for further research.

## 2. Epidemiology

Behçet's disease is mainly seen along the ancient Silk Road which stretched from the Far East to the Mediterranean Sea but has an occurrence worldwide. Turkey has the highest prevalence with studies reporting wide prevalence rates between 602 and 20 per 100,000 population, followed by Iran with 68 per 100,000 population (Table 1) [5–26]. The frequency increases in a north-to-south manner within the European continent [9, 27]. In Northern Europe, the prevalence ranges from 0.64 to 4.94 per 100,000 population [5–8]. It is noted that the prevalence has increased over time, which may relate to better awareness and/or migration [23, 24].

Previously thought to have an equal sex distribution, BD appears to have a male preponderance in Turkey [13, 14, 16], the Middle East, and Central Europe [28]; however, females predominate in Northwest Europe [5, 6] and the Far East [24–26, 29, 30]. Certain regional/geographical differences may exist; for example, pathergy phenomenon and ocular lesions are less commonly seen in the West [31–33], while gastrointestinal manifestations are more commonly seen in the Far East [34].

## 3. Etiology

The cause of BD remains largely unknown but it has been postulated that when a genetically predisposed or susceptible population is exposed to undetermined exogenous agents, this triggers dysregulation of both autoinflammatory and autoimmune responses resulting in multisystem vasculitis with distinct clinical characteristics. However, so far, no microbiologic or external environmental exposure has been consistently identified as a risk factor or trigger [35, 36]. More recently, dysbiosis of gut microbiota characterized by the reduction of the microbiota diversity and composition has been implicated in several autoimmune disorders including diseases outside of the gut [37, 38], and this dysbiosis seems to play a role in the pathogenesis of BD [39, 40].

Association of HLA-B^*∗*^51 allele is well recognized as the strongest genetic susceptibility gene so far among genetically predisposed BD patients [41, 42]. However, some studies have failed to demonstrate linkage [6, 42, 43], while certain indigenous Amerindians have a high prevalence of HLA-B^*∗*^51, but with no reported cases of BD [44]. A high level of recombination within the MHC is known to have occurred in these eastern populations before their migration. It was suggested that the disruption of genetic loci in linkage disequilibria within HLA-B^*∗*^51 might be one reason for the absence of disease in these high HLA-B^*∗*^51-bearing populations [44].

Centromeric regions including the tumour necrosis factor (TNF) gene and the MHC class I chain-related gene A (MICA) polymorphisms have been the focus of considerable research, particularly since TNF exerts a profound effect on the immune response, and MICA has a putative role in nonclassical antigen presentation at mucosal surfaces. However, it has become apparent that polymorphic areas in these regions, which are associated with BD, are in fact raised as a consequence of linkage disequilibrium within HLA-B^*∗*^51 and may provide little independent contribution to disease in HLA-B^*∗*^51-negative individuals. Thus, in most populations the highest risk factor for BD is still in or close to the HLA-B^*∗*^51 region [44].

## 4. Immunopathogenesis

Evidence suggests that a divergent and complex series of interactions and interplay between different cytokines, chemokines, and various components of the host immune system is involved in the pathogenesis of BD [45, 46]. There are several established cytokines such as TNF-*α*, interferon-gamma (IFN-*γ*), interleukin-1 beta (IL-1*β*), IL-6, IL-10, IL-17, and IL-23 known to be involved [45–47]. Furthermore, several “novel” cytokines are now implicated in the pathogenesis of BD including IL-2, IL-12, IL-21, IL-22, IL-33, and IL-37 [45, 48–59]; however, these require further studies to support a definitive role. Some of these findings have been replicated in the genome-wide association studies (GWAS) [60–64]. Moreover, the successful use of several anticytokine therapies in BD patients has provided additional evidence that cytokines play a crucial role in its pathogenesis [45, 46].

There is emerging evidence to support upregulation of chemokines in patients with BD [65–69], and a number of studies have implicated regulatory T cells (Tregs) and gammadelta (*γδ*) T cells in the immunopathogenesis of BD [70–73]. In addition, studies have also supported a role for neutrophil hyperfunction, endothelial cell activation [74–76], and activation of inflammasomes-dependent [77, 78] and JAK/STAT pathways [79, 80], while other studies have demonstrated correlations between autoantibodies such as anti-saccharomyces cerevisiae antibodies (ASCA) and anti-endothelial cell antibodies (AECA) and BD [81–86].

## 5. Clinical Manifestations

Commonly the symptoms of BD are self-limiting and tend to relapse in an unpredictable manner with differing phenotypes presenting among individuals but certain manifestations such as ocular, vascular, and neurological manifestations may lead to significant morbidity and death. Ideguchi et al. in their study in a Japanese population found that the time between the initial symptoms to the time of diagnosis was 8.6 years [87].

Oral aphthosis is the most common clinical feature and is usually the first manifestation [33]. It is characterized by round to oval ulceration with a white or yellowish necrotic base and surrounded by erythematous halo [88, 89]. It may involve any part of the oral mucosa, frequently the lips, buccal mucosa, tongue, gingiva, palate, and tonsils [33] and can be induced by local trauma such as after dental treatment (mucosal pathergy equivalent) [89]. The rate of recurrence and duration can vary with each attack and the majority of episodes are significantly painful [89]. Genital ulceration is the second most common observed feature of BD [33] and can occur on the scrotum, prepuce, glans, and shaft and tip of the penis in men, while it is typically seen on the vulva (labia majora, labia minora, and mons pubis) and/or intravaginal and cervical areas in women. They can also occur on the perianal, perineal, and groin areas. The ulcers are usually well defined, deep, and often painful and heal slowly with the larger and deeper lesions frequently healing with scarring [89].

The eye is the most common vital organ involved in BD [90] and over two-thirds of patients will develop ocular inflammation, most often bilateral panuveitis or retinal vasculitis [91]. Ocular manifestations include nongranulomatous iridocyclitis, chorioretinitis, or residual lesions suggesting previous iridocyclitis or chorioretinitis such as posterior synechia, complicated cataracts, lens pigmentation, chorioretinal atrophy, optic nerve atrophy, and secondary glaucoma. The prognosis is worse among patients with posterior segment involvement. Hypopyon in BD classically is nonsticky, forms a* niveau,* and tends to shift according to the head positioning [92]. Vision loss develops and worsens with each uveitis attack.

Skin manifestations are also one of the most common features of BD and these include but are not limited to erythema nodosum like eruptions, pseudofolliculitis, and papulopustular lesions. Despite being considered as one of the milder symptoms, skin manifestations may contribute to significant morbidity and impact negatively on patients' quality of life. Vascular BD affects both arteries and veins of all sizes and is more common in men than women [93]. Thrombophlebitis affecting superficial or deep veins is the most common manifestation, while arterial disease is less frequent but a major cause of mortality in BD patients. Neurological manifestations are relatively uncommon and can be classified into parenchymal (an inflammatory meningoencephalitic process with presence of isolated brainstem atrophy as a powerful discriminator) or nonparenchymal (secondary to vascular involvement) [94]. Diagnosis is difficult and often MRI brain (including contrast and MR venogram) and cerebrospinal fluid (evidence of neutrophilia and/or pleocytosis, frequently absent oligoclonal bands, and normal glucose levels) may assist in the diagnosis [94]. Other known manifestations include arthritis/arthralgia, cardiac, gastrointestinal, and laryngeal involvement [33, 95].

Pathergy phenomenon is a nonspecific cutaneous hyperreactivity response to minor trauma. There is large geographical variability in the prevalence of a positive pathergy test reaction [33, 96], and a decline in the positive rate over time has been detected [33, 97]. Positive pathergy testing is as high as up to 77% among patients in the Middle East, around the Mediterranean and the Far East [33, 98], but is less common in Northern European countries and the USA [43, 99–101].

## 6. Classification Criteria

The diagnosis of BD is clinical but not all symptoms occur simultaneously and the evolution varies among patients as well as among cohorts from different geographical areas. Furthermore, there is no laboratory test that can be used to make the diagnosis, hence the need for developing diagnostic disease criteria. The first diagnostic criteria in BD were devised in 1946 [102, 103], and now there are at least 17 diagnostic criteria available [103].

In 1990, the International Study Group (ISG) set the classification criteria [104] which were presented at the 6th International Conference on BD in Paris (1993) where recurrent oral aphthosis three or more times in a year is mandatory, with the presence of any two of the following: genital ulceration, ocular or cutaneous manifestations, or skin pathergy. It is important to note that the criteria are applicable only if no other clinical explanation is present. Despite being of high specificity and recognizing its contribution to assisting comparison among cohorts across the world in a more standardized manner, the criteria raised several important issues including the exclusion of minority groups of likely BD patients without the oral aphthosis and demonstrating relatively lower sensitivity in comparison to other diagnostic criteria [105].

To overcome these issues, in 2004 during the 11th International Conference on BD in Antalya, Turkey, The International Team for the Revision of the ISG criteria was formed involving 27 countries for the revision, proposal, and creation of the newer international criteria for BD (ICBD), which was then revised in 2010 [103]. These criteria are based on a point basis, oral aphthosis is not mandatory, and vascular and neurological manifestations were added to the existing five items of the ISG criteria. While oral aphthosis, genital ulceration, and eye manifestations were given two points, other remaining items were given one point each; getting four or more points confirms the diagnosis. It demonstrates improved sensitivity (97% versus 77.5%), a similar specificity (97% versus 99%), and better accuracy (97 versus 87%) when compared to the ISG criteria [103].

## 7. Current Management Strategies

Effective long-term management in BD patients is often challenging and requires a coordinated multidisciplinary approach. The cornerstone of treatment in systemic BD includes corticosteroids together with steroid-sparing agents (conventional immunomodulators and/or biological therapies) tailored upon the pattern and severity of patient's symptoms, mainly to achieve rapid resolution of inflammatory attacks, prevention of relapsing episodes, preservation of vital organs, and overall improvement in patients' quality of life.

### 7.1. Conventional Treatments

#### 7.1.1. Corticosteroids

Topical steroid therapy such as triamcinolone oral paste with or without topical anesthetics has been shown to be very useful for oral aphthosis in BD [106, 107]. Short courses of systemic oral or depot corticosteroids are particularly useful and effective in oral aphthosis resistant to topical treatment [108, 109] and controlling erythema nodosum especially in female BD patients [110]. High-dose pulsed intravenous methylprednisolone is reserved for patients with threatened vital-organ function. The European League Against Rheumatism (EULAR) task force recently in June 2016 presented their updated recommendations for managing BD at the 17th Annual European Congress of Rheumatology in London and recommended systemic corticosteroid as a combination therapeutic option in inflammatory ocular, vascular (including arterial aneurysms and acute deep vein thrombosis), gastrointestinal, or nervous system involvement [111]. Prolonged and frequent use of systemic corticosteroid however is associated with various unfavorable side effects.

#### 7.1.2. Colchicine

Being one of the oldest known drugs, colchicine has been proven to be beneficial in several randomized controlled trials in the less severe manifestations of BD patients including arthralgia, erythema nodosum, and genital ulcers [112, 113]. The EULAR task force in 2016 also recommended colchicine as first-line treatment for arthritis in BD patients [111]. Its early use however does not decrease the need to use immunosuppressive drugs in the long term [114].

#### 7.1.3. Conventional Immunomodulators

Frequently the additional use of a conventional immunomodulator as adjunctive or as steroid-sparing therapy is required. This is of utmost importance especially in countries with limited access to biological therapy. Agents such as methotrexate, azathioprine, mycophenolate mofetil, and cyclophosphamide have been shown to be beneficial [115–121] to induce and maintain remission. Cyclosporine and thalidomide are also options; however, cyclosporine has been strongly associated with neurological complications [122, 123], while thalidomide is highly neurotoxic and teratogenic and should be used with extreme caution if at all in women of childbearing age now that more modern therapies are available [124].

### 7.2. Biological Therapy

Emerging insights into the immunopathogenesis of BD have led to novel and more specific therapeutic targets. The advent of biological therapies has revolutionized the treatment of BD offering more tailored therapies resulting in significantly better disease control and prolonged remission in the majority of patients. This is an area that is rapidly expanding and is currently being robustly explored by both clinicians and researchers across the globe.

*Anti-TNFs* have been shown to be remarkably effective and relatively safe [125, 126]. Nonetheless, despite two decades of experience in rheumatological conditions such as BD, there are still many debatable issues surrounding their use including the following: (1) When to start treatment and the duration of treatment and does early use modulate the subsequent clinical course? (2) Is it beneficial to coadminister conventional nonbiologic DMARDs such as methotrexate to reduce immunogenicity and to reduce secondary failure rates? (3) The infection rates for combination therapy are especially high in elderly patients. (4) How effective are the “newer” anti-TNFs such as certolizumab pegol and golimumab? (5) Head-to-head studies to compare the safety and efficacy of different groups of biological agents, especially anti-TNF and interferon-2-alpha as monotherapy or in combination with conventional immunomodulators, are required.

Interferons (IFNs) are the oldest known cytokine and type-I IFN has been used as one of the treatment modalities in BD as early as the mid-1980s [127, 128]. There is gathering evidence, especially in more recent times, documenting the successful use of both* IFN-α-2a and -2b* with acceptable toxicity profiles in many clinical trials [129–133]. Contrary to IL-1*α*, IL-1*β* is not present in cells from healthy individuals [134] and has been demonstrated to be one of the principal highly active proinflammatory cytokines involved in the pathogenesis of BD [45–47]. There is emerging theoretical evidence for the use of* IL1β*-regulating antibody agents such as Gevokizumab [135, 136], Anakinra [137–140], and Canakinumab [141–144] in BD, and another orphan drug from this group Rilonacept has been successfully used in other autoinflammatory syndromes [145, 146].

Despite looking promising in the treatment of neuro-BD [147–152], the use of IL-6 blockade-*Tocilizumab* has yielded less convincing results than anticipated [152–155] and a controlled clinical trial for further evaluation of this biological agent has been terminated due to low enrollment [156]. Another humanized monoclonal antibody of IgG1 CAMPATH-1H-*Alemtuzumab* has been successfully used in refractory BD [157–159]. Other biological agents that are currently or have previously been considered worthy of consideration in the treatment of BD include the IL-12/23 monoclonal antibody-*Ustekinumab* [160, 161], B-cell depletion antibodies-*Rituximab* [162–166],* Belimumab,* and the competitive binding to CD80 and CD86 costimulator antibody-*Abatacept* which is currently undergoing an open-label clinical trial in the treatment of mucocutaneous manifestations of BD [167].

There are also “less successful” biological agents in BD: the IL-17A blockade by subcutaneous (SC)* Secukinumab* therapy failed to meet its primary endpoints in three randomized controlled clinical trials which involved 118 Behçet's uveitis patients [168]; however, Letko et al. [169] argued that the dose and the mode of administration may have been a confounding factor and suggested in their proof-of-concept study a higher dose and a different route of administration of Secukinumab (from 300 mg SC to intravenous administration of 30 mg/kg and 10 mg/kg). Another monoclonal antibody* Daclizumab*, an IL-2 receptor antagonist, also failed to demonstrate efficacy in Behçet's uveitis patients compared to placebo [170] despite demonstrating potent efficacy in many previous studies for other causes of noninfectious uveitis [171–173], while evidence for treatment with intravitreal* Bevacizumab*, a vascular endothelial growth factor A antibody to treat inflammatory ocular manifestations, has been conflicting [174–178].

### 7.3. Novel Nonbiologic Small Molecules

Apremilast, a novel small molecule that selectively inhibits phosphodiesterase 4 (PDE4) which is currently approved for psoriasis and psoriatic arthritis, has been shown in a randomized, double blind, placebo-controlled phase 2 study to be effective in treating oral ulcers in BD patients [179]. It is currently undergoing subsequent phase 3 trials. The demonstration of Janus kinase-1/signal transducers and activators of transcription-3 (JAK1/STAT3) signaling pathway activation in BD [79] suggests a potential role for JAK inhibitors (Jakinibs) as a possible next-generation therapeutic modality in the management of BD.

## 8. Controversies, Conundrums, and Chasms: Prospects for Further Research

BD has been the subject of extensive investigation since its first formal description approximately 80 years ago. Despite best efforts, controversies continue to exist and several questions in many aspects of BD remain unanswered. The 17th International Conference on BD held in Matera, Italy, in September 2016 was a unique opportunity to reflect on the residual challenges that remain in BD and also to highlight new advances in research from the scientific community across the world.

The main areas where controversies continue to exist relate to (1) diagnostic criteria, (2) immunopathogenesis and the search for biomarkers, (3) regional and geographical phenotypic and genotypic variability, and (4) therapeutic considerations including the use of biological therapies and the role of anticoagulation in thrombosis.

One of the major challenges still faced by clinicians is the diagnostic dilemma due to the wide spectrum or heterogeneity of disease manifestations with varying severity, the unpredictable relapsing and remitting episodes of most patients, and the variable chronological evolution of symptoms between different individuals. Despite recognizing mucocutaneous lesions as the hallmark of the disease with ocular inflammation and skin lesions considered part of the major symptoms, a discreet subset of patients manifests other less common yet important characteristics such as vascular, neurological, gastrointestinal, and laryngeal disease. Besides the controversy regarding the optimal management approach among this discreet subset of patients, these infrequent manifestations may lead to significant delay in diagnosis resulting in irreversible organ damage for the patient.

A closely related issue that remains a research enigma is the lack of sensitive and specific diagnostic laboratory tests to confirm or support the final diagnosis of BD. Until we discover such biomarkers, the burden of diagnosis remains with the clinician's ability to recognize and collate a diverse spectrum of presenting manifestations. A substantial number of patients with BD remain undiagnosed for many years resulting in a significant increase in morbidity, disability, and worsening quality of life.

One possible solution to this dilemma is to broaden the classification criteria combining both objective clinical indicators and biomarkers. However, despite the emergence of a number of potential candidate biomarkers, there is still a lack of sufficient evidence to support their implementation and incorporation into the contemporary classification criteria. In the era of precision medicine, this area provides a significant opportunity for improvement in the diagnostic criteria and to find early predictors to detect cohorts with a severe aggressive disease phenotype. Better understanding of disease pathways and the continuous search for signature markers may provide novel insights into early detection of the disease in the future and providing potential targets for novel therapeutic agents so patients may be treated with the best treatment option in a timely manner.

One of the groundbreaking discoveries of late is the recognition that BD may be diagnosed solely based on ocular findings alone in the absence of systemic manifestations [90] using the state-of-the-art imaging technologies, and this is extremely important as ocular manifestation may be the first presenting manifestations in 10–15% of BD patients [180]. The ultimate goal in eye disease is to sustain remission with preservation of vision [91]. Recent studies support earlier and more frequent consideration for biological therapy in Behçet's uveitis [91] but questions remain regarding when to use them in patients with uveitis alone and with classical uveitis characteristics but not fulfilling the international criteria for BD. Steroids, while able to rapidly control acute flares, are not able to reduce recurrence rates and their prolonged use is associated with serious side effects.

Recent attention has also focused on the presence of racial, geographical, or regional predilection in phenotypic heterogeneity and genetic variance. Despite some well-documented evidence from endemic areas, the advancement in epidemiological understanding in nonendemic areas especially Northern European countries has been particularly difficult. This is probably one of the most perplexing problems and reflects a lack of detailed epidemiological studies in the so-called nonendemic regions across the globe.

BD is generally described as a polygenic disease; however, family clustering in BD has been described in the literature [181–185]. There is an aberrant subset of BD that carries autosomal-dominant traits highlighting a different pathway in disease pathogenesis. Several candidate gene mutations have been discovered so far including MEFV/TLR4 mutations [186] and more recently TNFAIP3 mutations [187, 188] suggesting several different possible underlying mechanisms to induce inflammation from these mutations including the more recent concept of haploinsufficiency of A20 (HA20) [189]; however, data are still scarce and limited. Targeted next-generation exome sequencing which has the ability to generate millions of short reads of sequence within a short period of time looks promising in novel gene discovery and may provide answers to many questions in the future including how broad these spectrum of disorders are and if there are many other mutations that can cause similar phenotypic picture.

While nonbiological DMARD agents such as thalidomide, methotrexate, azathioprine, and cyclosporine may provide some benefit to these patients, they carry a higher side-effect profile. In this cutting edge era, interferon alfa-2a and TNF have been proven in many instances to be more effective and safer. Newer treatment paradigms showing promising results include ustekinumab, canakinumab, apremilast, tocilizumab (especially in neuro-BD), and brodalumab (for severe mucocutaneous manifestations) and many are undergoing clinical trials. Gevokizumab despite failing to achieve its primary endpoint, which was time to a first ocular exacerbation in a phase III study, may still be effective in preservation of visual acuity, inducing less severe exacerbations and lower incidence of macula edema, and is currently undergoing 2 further trials. However, access to biological agents may be the limiting factor in many countries and it will be important to identify patients who may be resistant to certain therapies and those who will benefit the most from a particular intervention. Head-to-head trials involving the newer and most current agents rather than placebo-controlled should be undertaken in patients with systemic disease or with vital-organ involvement due to the known grave irreversible consequences in untreated or inadequately treated patients.

A question that has been long debated is whether or not to anticoagulate patients with vascular thrombosis. So far, the decision regarding the use of concomitant anticoagulation with the more definitive glucocorticoids and immunosuppressive treatment is based upon individual consultant opinion and retrospective studies [190]. Due to the paucity of evidence, and until randomized controlled trial tests the efficacy of anticoagulation strategies, this question remains unanswered [190]. Caution is needed especially in the rare yet lethal condition known as Hughes-Stovin syndrome where patients have a combination of pulmonary artery aneurysm and deep vein thrombosis. Immunosuppressive therapy remains the mainstay of treatment in vascular BD to induce remission, prevent further relapse, and improve patients' survival.

Pregnancy can pose a major challenge in BD as the evidence regarding the effect of BD on pregnancy and vice versa is limited. The disease course varies and is difficult to predict during pregnancy [191–193]. Despite a study demonstrating a lower proportion of flares in pregnant BD patients treated with colchicine [194] and another study observing no increased rate of pregnancy-related complications in BD patients [194, 195], several other studies have demonstrated higher miscarriage rates [196, 197], caesarian section rates [196], and smaller babies [197] in BD patients. Other potential issues should also be addressed in pregnancy and in particular the management of pregnant BD patients with known thrombotic tendencies.

In this review, we highlight recent advances in our scientific understanding of BD and shed new insights into diagnostic and treatment strategies. Despite the increase in published scientific literature on BD and the growing interest of a global research community, many aspects of BD remain enigmatic and controversial. Ironically, these deficits in our knowledge serve as a stimulus and challenge to the global scientific community to seek answers to these research questions through national and international collaborations. There is a pressing need for international epidemiological studies of BD with geographical and ethnic mapping, more basic science discoveries to unravel the complex immunopathogenesis, innovation in biomarker discovery to improve diagnostic yield, and large-scale randomized controlled trials to assess therapeutic benefit of current and emerging therapies. Behçet's disease,* the Silk Road disease,* has challenged the scientific and clinical communities to come together with a cohesive strategy to foster greater understanding of this rare disease in order to improve patient outcomes.

## Figures and Tables

**Table 1:** The worldwide prevalence of BD.

Country/area	Study (year)	Patients (*n*)	Population/ participants	Prevalence (per 100,000)
Northern Europe				
UK (Yorkshire)	Chamberlain (1977) [5]	32	5,000,000	0.64
Scotland	Jankowski et al. (1992) [6]	15	5,500,000	0.3
Ireland (Dublin)	Kilmartin et al. (1997) [7]	24	1,058,264	2.27
Sweden (Skane)	Mohammad et al. (2013) [8]	40	809,317	4.94
Europe				
Germany	Papoutsis et al. (2006) [9]	165	3,391,344	4.87
France (Paris)	Mahr et al. (2008) [10]	79	1,094,412	7.1
Italy (Rome)	Valesini et al. (1991) [11]	155	NA	19
Greece	Kaklamani et al. (2000) [12]	90	NA	11
Mediterranean				
Turkey				
Tokat	Baş et al. (2016) [13]	14	2,325	602
Kayseri	Çölgeçen et al. (2015) [14]	9	5,218	170
Havsa	Cakir et al. (2004) [15]	1	4,861	20
Istanbul	Azizlerli et al. (2003) [16]	101	23,986	420
Ankara	Idil et al. (2002) [17]	16	17,256	110
Ordu	Yurdakul et al. (1988) [18]	19	5,121	370
Istanbul	Demirhindi et al. (1981) [19]	4	4,940	80
Mediterranean				
Iran	Davatchi et al. (2007) [20]	7	10,291	68
Egypt	Assaad-Khalil et al. (1997) [21]	274	NA	16
Jordan	Madanat et al. (2000) [22]	200	NA	27
Others				
US (Olmsted County)	Calamia et al. (2009) [23]	13	144,248	5.2
Japan	Nakae et al. (1993) [24]	3316	NA	13.5
Korea	Bang et al. (2011) [25]	15,554	NA	30.2
China	Zhang et al. (2006) [26]	1996	NA	14
